# Regional brain iron and gene expression provide insights into neurodegeneration in Parkinson’s disease

**DOI:** 10.1093/brain/awab084

**Published:** 2021-03-11

**Authors:** George E C Thomas, Angeliki Zarkali, Mina Ryten, Karin Shmueli, Ana Luisa Gil-Martinez, Louise-Ann Leyland, Peter McColgan, Julio Acosta-Cabronero, Andrew J Lees, Rimona S Weil

**Affiliations:** 1 Dementia Research Centre, UCL, London, WC1N 3AR, UK; 2 Department of Neurodegenerative Disease, UCL Institute of Neurology, London, WC1B 5EH, UK; 3 NIHR Great Ormond Street Hospital Biomedical Research Centre, UCL, London, WC1N 1EH, UK; 4 Genetics and Genomic Medicine, Great Ormond Street Institute of Child Health, UCL, London, WC1N 1EH, UK; 5 Department of Medical Physics and Biomedical Engineering, Malet Place Engineering Building, UCL, London, WC1E 6BT, UK; 6 Huntington’s Disease Centre, UCL Institute of Neurology, London, WC1B 5EH, UK; 7 Tenoke Ltd., IdeaSpace South, Cambridge Biomedical Campus, Cambridge, CB2 0AH, UK; 8 Reta Lila Weston Institute of Neurological Studies, London, WC1N 1PJ, UK; 9 Wellcome Centre for Human Neuroimaging, UCL, London, WC1N 3AR, UK; 10 Movement Disorders Consortium, UCL, London, WC1N 3BG, UK

**Keywords:** Parkinson’s disease, iron, quantitative susceptibility mapping, genetics, transcriptomics

## Abstract

The mechanisms responsible for the selective vulnerability of specific neuronal populations in Parkinson’s disease are poorly understood. Oxidative stress secondary to brain iron accumulation is one postulated mechanism. We measured iron deposition in 180 cortical regions of 96 patients with Parkinson’s disease and 35 control subjects using quantitative susceptibility mapping. We estimated the expression of 15 745 genes in the same regions using transcriptomic data from the Allen Human Brain Atlas. Using partial least squares regression, we then identified the profile of gene transcription in the healthy brain that underlies increased cortical iron in patients with Parkinson’s disease relative to controls. Applying gene ontological tools, we investigated the biological processes and cell types associated with this transcriptomic profile and identified the sets of genes with spatial expression profiles in control brains that correlated significantly with the spatial pattern of cortical iron deposition in Parkinson’s disease. Gene ontological analyses revealed that these genes were enriched for biological processes relating to heavy metal detoxification, synaptic function and nervous system development and were predominantly expressed in astrocytes and glutamatergic neurons. Furthermore, we demonstrated that the genes differentially expressed in Parkinson’s disease are associated with the pattern of cortical expression identified in this study. Our findings provide mechanistic insights into regional selective vulnerabilities in Parkinson’s disease, particularly the processes involving iron accumulation.

## Teaser

Oxidative stress secondary to brain iron accumulation has been implicated in extrastriatal neurodegeneration in Parkinson’s disease. Thomas et al. show that cortical iron deposition in Parkinson’s disease is associated with differences in the expression of genes involved in metal detoxification and synaptic function.

## Introduction

In Parkinson’s disease, bradykinesia and rigidity severity correlate approximately with the degree of nigrostriatal dopamine denervation^1^ and the presence of α-synuclein-rich cell inclusions,^2^ Lewy bodies, which represent the pathological signature associated with the disease. However, α-synuclein inclusions do not correlate with symptom severity^3^ and the pathophysiological processes that are responsible for the associated dementia seen in many elderly patients remain unknown. As such, there is an increasing need to understand why some brain regions display selective vulnerability to neurodegeneration^4^ and determine the mechanisms that trigger the degenerative cascade.^5^

For over 25 years, it has been known that iron accumulates in the basal ganglia and substantia nigra in Parkinson’s disease^6-8^ with the resultant oxidative stress being of interest as an important potential driver of neurodegeneration.^9–11^ Moreover, this observation has been corroborated by recent advances in the study of this disease.^12^^,^^13^ Iron accumulates in the brain, especially within the basal ganglia, during ageing,^14^ partly due to the increased permeability of the blood–brain barrier.^15^ High levels of iron in the tissue cause a build-up of toxic reactive oxygen species that interfere with mitochondrial function,^16^ damage DNA,^17^ catalyse dopamine oxidation reactions to produce toxic quinones^18^ and irreversibly modify proteins through highly reactive aldehydes.^19^ All these causes of cell stress ultimately lead to iron-mediated cell death.^20^ Intriguingly, lipofuscin formation, previously characterized in Parkinson’s disease,^21^ has recently been shown to be driven by the disruption of lysosomal lipid metabolism in neurons.^22^ This in turn leads to iron accumulation, oxidative stress and ferroptosis,^22^ providing a direct link between the pathological inclusions in Parkinson’s disease and excess brain iron. Excess iron also interacts directly and indirectly through free radical species with key pathological proteins associated with Parkinson’s disease by promoting the aggregation of α-synuclein,^23^ stimulating the production of amyloid-β via the downregulation of furin^24^ and increasing the toxicity of amyloid-β either directly^25^ or through increased tau phosphorylation.^26^

Until recently, whole brain neuroimaging using conventional structural MRI has maintained only a limited role in uncovering neurodegenerative mechanisms in Parkinson’s disease as cortical atrophy is not prominent, particularly early in the disease process.^27^ However, quantitative susceptibility mapping (QSM)^28^ has emerged as a powerful new technique, which is sensitive to local sources of magnetic susceptibility and in particular to variation in brain iron content.^29^^,^^30^ We recently used QSM to show that brain iron is associated with disease severity in Parkinson’s disease,^31^ with higher levels of hippocampal iron linked to poorer cognition and higher putaminal iron linked with worse motor performance. Whether brain iron deposition is a cause of neuronal damage or a surrogate marker remains unknown, but QSM offers a new way to explore the regional effects of neurodegeneration.

We have used the spatial information about iron accumulation provided by QSM and combined it with regional gene expression profiles from the Allen Human Brain Atlas transcriptomic data^32^ to identify possible mechanisms for regional selective vulnerability in Parkinson’s disease. The approach of combining neuroimaging with transcriptomic data has previously been used to investigate autism,^33^ schizophrenia^34^ and Huntington’s disease.^35^ Here, we used partial least squares (PLS) regression to test whether cortical brain iron accumulation in Parkinson’s disease measured using QSM correlates with specific patterns of gene expression to shed light on the gene expression profiles that render specific cortical regions vulnerable to higher levels of brain iron accumulation and subsequent neurodegeneration. We also performed an enrichment analysis on the identified gene expression patterns linked to higher brain iron to determine the biological and cell processes linked with the degenerative process of aberrant iron accumulation and neurodegeneration.

## Materials and methods

### Participants

Ninety-six patients with Parkinson’s disease with a disease duration of <10 years [age 49–80 years, mean = 66.4, standard deviation (SD) = 7.7, 48 females] volunteered to participate from October 2017 to December 2018. The inclusion criteria were a clinical diagnosis of early to mid-stage Parkinson’s disease (Queen Square Brain Bank Criteria^36^) in the age range of 49–80 years. Exclusion criteria were confounding neurological or psychiatric disorders, dementia and metallic implants considered unsafe for MRI. Participants continued their usual therapy (including l-DOPA) for all assessments. No patients were taking cholinesterase inhibitors. In addition, we recruited a group of 35 age-matched controls (50–80 years, mean = 66.1, SD = 9.4, 21 females) that included some unaffected patient spouses. All participants gave written informed consent, and the study was approved by the Queen Square Research Ethics Committee. All participants underwent clinical assessments of motor function, cognition, vision, mood and sleep, as previously described.^31^ Motor assessments were performed using the Movement Disorder Society-Unified Parkinson’s Disease Rating Scale,^37^ general cognition was assessed using the Mini-Mental State Examination^38^ and the Montreal Cognitive Assessment^39^ (see Table 1 for clinical and demographic information).

**Table 1:** Participant demographics

Measure	Control	Parkinson’s disease	*P*-value
	(*n *=* *35)	(*n *=* *96)	
Gender, male: female	15:20	51:45	0.329
Age, years	66.26 (9.16)	64.52 (7.79)	0.284
Years of education	17.71 (2.38)	17.02 (2.83)	0.196
MoCA (out of 30)	28.71 (1.34)	27.97 (2.06)	0.049^*^
UPDRS-III	5.20 (4.21)	22.25 (11.46)	2.66 × 10^−14***^
Binocular LogMAR visual acuity	−0.08 (0.23)	−0.09 (0.13)	0.802
HADS depression score	1.66 (1.89)	3.81 (2.83)	5.37 × 10^−5***^
HADS anxiety score	3.91 (3.43)	5.92 (4.07)	0.011^**^
RBDSQ score	1.94 (1.39)	4.13 (2.46)	2.17 × 10^−6***^
Smell test (Sniffin’ Sticks)	12.49 (2.43)	7.63 (3.13)	1.08 × 10^−13***^
Disease duration, years	N/A	4.20 (2.52)	N/A
Levodopa equivalent dose, mg	N/A	459 (256)	N/A
Motor deficit dominance, left:right:both	N/A	37:54:5	N/A

Means (SD) are reported. Binocular LogMAR: lower score represents better visual acuity. HADS = Hospital Anxiety and Depression Scale; MoCA = Montreal Cognitive Assessment; RBDSQ = REM Sleep Behaviour Disorder Screening Questionnaire; UPDRS = Unified Parkinson’s Disease Rating Scale. ***P <* *0.001; **P < 0.01; *P < 0.05; ns = not significant.

### MRI acquisition

MRI acquisition was as previously described.^31^ In brief, MRI measurements consisting of susceptibility- and T_1_-weighted MRI scans were performed on a Siemens Prisma-fit 3-T MRI system using a 64-channel receive array coil (Siemens Healthcare). Susceptibility-weighted MRI signals were obtained from a 2 × 1-accelerated, 3-D flow-compensated spoiled-gradient-recalled echo sequence with flip angle, 12°; echo time, 18 ms; repetition time, 25 ms; receiver bandwidth, 110 Hz/pixel; matrix dimensions 204 × 224 × 160 with 1 × 1 × 1 mm voxel resolution (scan time, 5 min 41 s). T_1_-weighted magnetization-prepared 3-D rapid gradient-echo (MPRAGE) anatomical images were acquired using a 2 × 1-accelerated sequence with inversion time, 1100 ms; flip angle, 7°; first echo time, 3.34 ms; echo spacing, 7.4 ms; repetition time, 2530 ms; receiver bandwidth, 200 Hz/pixel; matrix dimensions 256 × 256 × 176 with 1 × 1 × 1 mm voxel size (scan time, 6 min 3 s).

### QSM preprocessing and spatial standardization

QSM preprocessing was as previously described.^31^ 3-D phase images were unwrapped with a discrete Laplacian method.^40^ Brain masks were calculated using the BET2 algorithm^41^ in the FMRIB software library (FSL version 5.0, https://fsl.fmrib.ox.ac.uk/fsl/fslwiki; accessed Jan 2019). Background field removal was completed using Laplacian boundary value extraction^42^ and variable spherical mean-value filtering,^43^ and susceptibility maps were estimated using Multi-Scale Dipole Inversion^44^ in MATLAB R2014b (The MathWorks, Inc., Natick, MA, USA). A study-wise template was created using advanced normalization tools^45^ via the normalization of participant MPRAGE volumes.^31^^,^^46^ QSM images were co-registered to normalized space using a warp composition of the above transformation and an affine gradient echo-magnitude-to-MPRAGE transformation using advanced normalization tools.

### Regional QSM analysis

**Figure 1:**
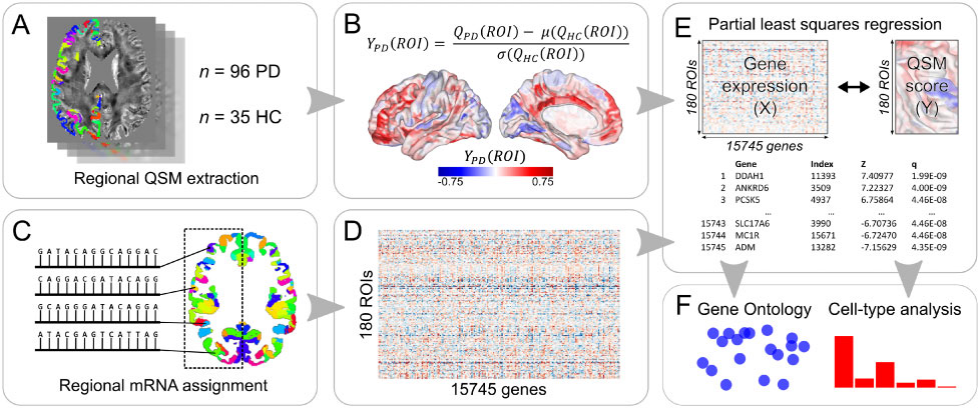
**An overview of the methodology used for regional QSM extraction, estimation of regional gene expression, PLS regression, gene ontological and cell type analyses.** (**A**) Mean, signed QSM values were extracted from 180 left-cortical regions for 96 patients with Parkinson’s disease and 35 controls. (**B**) A QSM score, Y_PD_, was calculated for each region by a *z*-score transformation. (**C** and **D**) Allen Human Brain Atlas samples of gene expression data were mapped to the 180 left-cortical regions according to the anatomical parcellation and were used to create a matrix containing the average expression of 15 745 genes in those regions. (**E**) A bootstrapped PLS regression was performed using gene expression (*X*) as the predictor variable and the QSM score (*Y*) as the response variable. The second component of *X* explained maximum variance in *Y* and bootstrapped *z*-scores were used to rank each gene’s contribution to this component. (**F**) Genes that were significant at *Q* (corrected *P*) < 0.05 underwent gene ontological analyses for biological processes and expression-weighted cell-type enrichment analyses.

In our previous whole-brain analysis, absolute QSM was used to improve statistical conditioning in the cortex.^14^ However, as the current study involved multiple individual regions of interest, we used signed QSM, as it enables discrimination between paramagnetic and diamagnetic susceptibility sources. To generate regions of interest, we used a version of the Glasser atlas surface parcellation^47^ transformed into a set of volumetric labels in MNI space (https://neurovault.org/collections/1549/; accessed February 2020). The 180 cortical regions of interest from the left hemisphere were brought into the study-wise template space using an advanced normalization tool-based deformable b-spline co-registration routine and nearest-neighbour interpolation. To reduce partial-volume contamination, each cortical region of interest was intersected with a study-wise average grey matter mask binarized at a grey matter density cut-off of 0.25 using FSL. Mean, signed QSM values in each region of interest were extracted from all participants and age- and sex-adjusted using MATLAB. A linear model was fitted to each region of interest in all the control subjects such that:

(1) $${\hat{Y}}_{i}=\;\alpha_{i}+\beta_{i}A$$

where ${\hat{Y}}_{i}$ is the estimated mean susceptibility value in region *i*, *A* is the subject’s age and *α_i_* and *β_i_* are the fitted model parameters. The QSM susceptibility values in the controls and patients with Parkinson’s disease were then age-adjusted according to:

(2) $$\chi_{ij}=Y_{ij}+\beta_{i}(\mu -A_{j})$$

where, for region *i* in subject *j*, *Y_ij_* is the unadjusted susceptibility value, *µ*, is the mean age of the control subjects, *A_j_* is subject’s age and *χ_ij_* is the age-adjusted susceptibility value. We used the fitted parameters from the control group to age-correct the susceptibility values measured in the patients with Parkinson’s disease to avoid removing effects of disease progression with age. For the sex adjustment, as sex may affect magnetic susceptibility values differently in controls and Parkinson’s disease patients, a linear model was fitted to each region of interest for each participant group separately:

(3) $${\hat{\chi }}_{ik}=\;\alpha_{ik}+\beta_{ik}S_{k}$$

where, for region *i* in participant group *k*, ${\hat{\chi }}_{ik}$ is the estimated age-adjusted susceptibility, *S_k_* is the sex of the subject and *α_i__k_* and *β_i__k_* are the fitted parameters. We then adjusted for the effect of sex in each group using:

(4) $${\chi '}_{\mathrm{ijk}}=\chi_{\mathrm{ijk}}+\beta_{ik}(\mu_{k}-S_{jk})$$

where, for region *i* in subject *j* in participant group *k*, *χ_ijk_* is age-adjusted susceptibility, *µ_k_* is the group’s ‘mean’ sex, *S_jk_* is the subject’s sex and *χ′_ijk_* is the age- and sex-adjusted susceptibility. To generate a continuous measure of magnetic susceptibility difference in patients with Parkinson’s disease relative to the controls, Parkinson’s age- and sex-adjusted regional means were normalized to the control mean for that region by a *z*-score transformation using MATLAB,^48^ giving a 180 × 1 age-adjusted QSM score vector, *Y* (see Fig. 1 for an overview of QSM and gene expression processing). Statistically significant differences between the regions of interest were probed using *t*-tests and are reported at *P* < 0.05 and *Q *< 0.05.^49^

### Estimation of regional gene expression

We used the Allen Human Brain Atlas microarray transcriptomic data for five male and one female donors with no history of neurological or psychiatric disease (mean age 42.5 years), available from the Allen Institute for Brain Science.^32^ As data were available from six donors for the left hemisphere but only two donors for the right hemisphere, we assessed the left hemisphere samples only, as these were considered more robust.^35^^,^^50^ Using MATLAB, each tissue sample was assigned to one of the 180 left cortical regions of the Glasser atlas,^47^ using the Allen Human Brain Atlas MRI data for each donor, in a process that has previously been described in detail.^48^ Genes with expression levels above a background threshold of 50% were selected and gene expression data were normalized across the left cortex, as previously described.^48^ Regional expression levels for each gene were compiled to form a 180 × 15 745 regional transcription matrix, *X* (Fig. 1).

### Partial least squares regression

We used PLS regression to examine the association between the healthy brain transcriptome and cortical QSM in Parkinson’s disease, as this technique is well suited to the high collinearity of gene expression data.^51^ PLS regression is a multivariate analysis technique, similar to principal component analysis, which combines dimension reduction and linear regression, producing components from *X* (the 180 × 15 475 predictor matrix of 180 regional mRNA measurements for 15 475 genes) that have maximum covariance with *Y* (the 180 × 1 regional QSM score vector). The second PLS component (PLS2) was used to weigh and rank gene predictor variables. In total, 10 000 permutations based on sphere-projection-rotations^52^ of the QSM-score cortical map were examined to test the null hypothesis that PLS2 explained no more variance in *Y* than chance. Bootstrapping was used to estimate the variability of each gene’s positive or negative weight on PLS2 and the ratio of the weight of each gene to its bootstrapped standard error was used to rank its contribution to the PLS2. All PLS and bootstrapping analyses were conducted in MATLAB. We tested the null hypothesis of zero weight for each gene using a false discovery rate inverse quantile transformation correction to account for winner’s curse bias using R version 3.6.1.^53^ Only genes that survived this correction at *Q* < 0.05 were included in the enrichment analyses, and upweighted and downweighted genes were assessed separately.

### Gene ontological analysis

We used the g:Profiler^54^ toolset, implemented in R, to perform a gene ontological (GO) enrichment analysis of the significant positively and negatively weighted genes defined by PLS2. We filtered the resulting list of GO terms by retaining only those that were significantly enriched at *P* < 0.05 (corrected for multiple comparisons using the g:SCS algorithm^54^) and discarded the terms associated with >2500 genes as being too general. To reduce and visualize the GO terms, we used the REViGO web page tool, which is based on semantic similarity.^55^

We performed additional GO enrichment analyses using R to mitigate against the possibility of false-positive bias for GO terms in the enrichment analyses of brain-wide transcriptomic data due to null models not accounting for gene-gene co-expression and spatial autocorrelation present within such data.^56^ Specifically, we ran GO enrichment analyses for both a random and spatial-spin permutation of our QSM score data (generated in MATLAB) and compared the associated GO terms to those arising from our main analysis.

### Cell-type analysis

As gene expression is often driven by the underlying cell-type distribution, we used expression-weighted cell-type enrichment analysis, implemented in R, to investigate whether the most strongly weighted genes were more significantly expressed in particular cell types.^57^ We took the top 20% of the most significantly upweighted and downweighted genes identified by PLS2 and assessed their relative expression in the cell types defined in the Allen Institute for Brain Science single-cell transcription dataset (https://portal.brain-map.org/atlases-and-data/rnaseq)^58^ against a background set of all 15 745 genes included in our initial analysis. We replicated our analysis using a separate human derived dataset^59^ and performed replication analyses with the top 10, 30, 40 and 50% of PLS2 genes to ensure that the results were not driven by the threshold selection.

### Comparison with external Parkinson’s disease post-mortem-derived gene expression data

To test whether differentially expressed genes in Parkinson’s disease and associated disease states help to explain the pattern of PLS2 expression, we matched the sets of genes identified in cortical^60–62^ and subcortical^61^^,^^63^^,^^64^ brain regions in Parkinson’s disease, as well as cortical regions in Parkinson’s disease dementia^62^ and dementia with Lewy bodies,^61^ to our weighted PLS2 gene list. This included the reanalysis of one dataset^60^ using the available clinical data to differentiate between Parkinson’s disease and Parkinson’s disease dementia, with the RNA-sequencing data in each group analysed using the R package DESeq2 version 1.30.0. We used age at death and the RNA integrity number as covariates and generated lists of genes that were differentially expressed in: Parkinson’s disease relative to controls; Parkinson’s disease dementia relative to controls; and Parkinson’s disease dementia relative to Parkinson’s disease (significant at *P* < 0.05). We tested whether genes in each dataset were significantly more positively or negatively weighted than was due to chance by using 10  000 random permutations of the same sample size. To maintain consistency across datasets, we only included genes with an absolute fold-change of >1.5. The significant results are reported at Bonferroni corrected *P *<* *0.0023 (22 comparisons across five studies).

### Data availability

The regional QSM scores and gene expression matrices, along with the code to carry out the PLS regression, gene ontological, and weighting of differentially expressed genes analyses can be found at (https://github.com/gecthomas/QSM_and_AHBA_transcription_in_PD; last updated 26 March 2021). Code for cortical parcellation of Allen Human Brain Atlas data into Glasser regions^48^: (https://github.com/BMHLab/AHBAprocessing; accessed February 2020). Code for creating spatial spin permutations of MRI data was adapted from Váša *et al*.^52^ (https://github.com/frantisekvasa/rotate_parcellation; accessed March 2020). Code for PLS analyses and bootstrapping was adapted from Whitaker *et al*.^65^: (https://github.com/KirstieJane/NSPN_WhitakerVertes_PNAS2016/tree/master/SCRIPTS; accessed March 2020). GO analyses were conducted using the g:Profiler2 R package (https://CRAN.R-project.org/package=gprofiler2; accessed October 2020). Cell-type analyses were conducted using the expression-weighted cell-type enrichment analysis R package^57^ (https://github.com/NathanSkene/EWCE; accessed July 2020). BrainNet Viewer was used to visualize data on cortical surfaces: (https://www.nitrc.org/projects/bnv/; accessed December 2019).^66^

## Results

### Brain iron content is increased in Parkinson’s disease compared with control subjects

**Figure 2:**
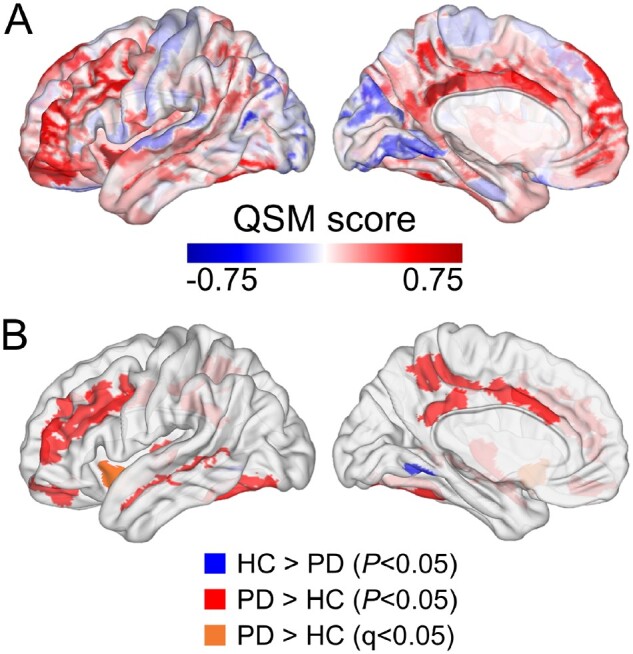
**Regional cortical differences in magnetic susceptibility between Parkinson’s disease and controls.** (**A**) The cortical plot of the QSM scores, calculated by *z*-score transformation of the Parkinson’s disease mean to the control mean for each of the 180 Glasser regions of interest. (**B**) Glasser regions of interest where a significant difference was observed [blue, controls greater than Parkinson’s disease at *P* < 0.05; red, Parkinson’s disease greater than controls at *P* < 0.05; orange, Parkinson’s disease greater than controls at *Q* (FDR-adjusted *P*) < 0.05].

**Figure 3:**
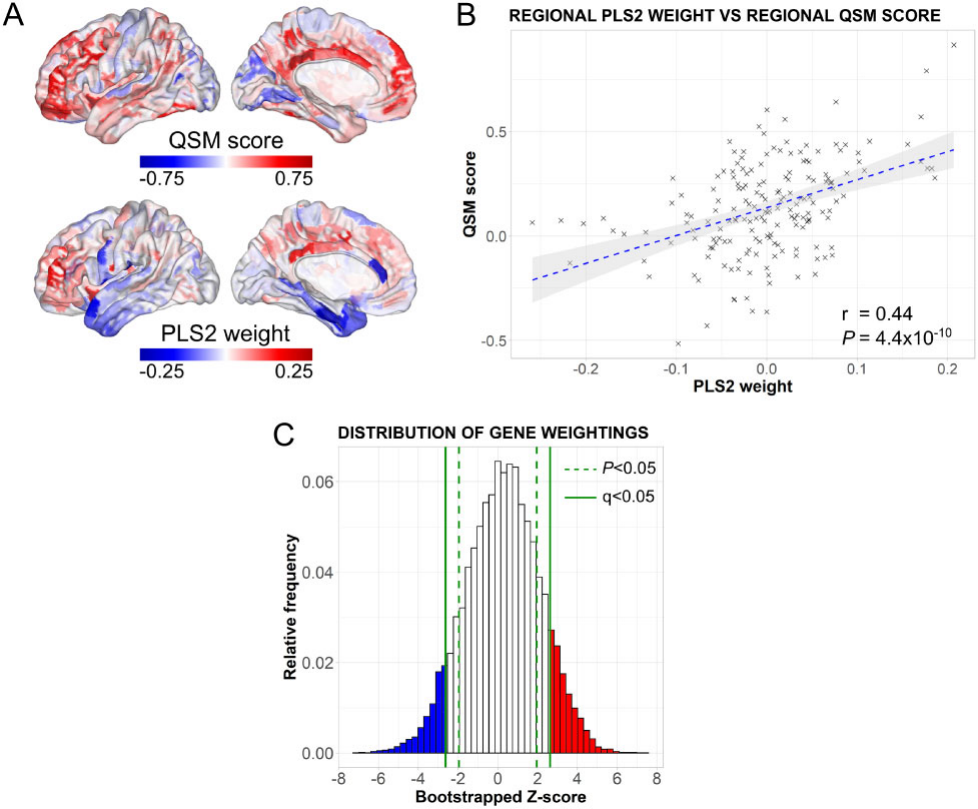
**Spatial profiles of gene expression and significantly weighted genes associated with cortical iron deposition in Parkinson’s disease.** (**A**) The cortical map of QSM scores had a similar spatial pattern to the regional linearly weighted sum of gene expression scores defined by the PLS2. (**B**) A scatterplot of regional PLS2 scores versus QSM scores demonstrating a positive correlation; each data point represents one of 180 cortical regions. (**C**) The distribution of bootstrapped gene weights on PLS2. An FDR inverse quantile transform was used to correct for multiple comparisons, giving a set of 1622 upweighted (red) and 1068 downweighted (blue) genes significant at *Q* (FDR inverse quantile transform-corrected *P*) < 0.05 that were used in gene ontological and cell-type analyses.

The regional cortical signed QSM scores showed increases in magnetic susceptibility in patients with Parkinson’s disease relative to controls in the frontal, posterior parietal and insular cortices, and slightly decreased susceptibility values in patients with Parkinson’s disease relative to controls in the occipital cortex (Figs 2A and 3B), indicating differences in brain iron content in these brain regions. These differences were statistically significant (*P* < 0.05) in 17 Glasser regions of interest, with increased magnetic susceptibility in Parkinson’s disease in 16 regions of interest and increased magnetic susceptibility in controls compared with Parkinson’s disease in one regions of interest (Fig. 2B and Table 2). The anterior agranular insular complex survived correction for multiple comparisons (FDR-adjusted *Q* < 0.05).^49^ These results were qualitatively similar to the whole-brain results we obtained using absolute QSM in our previous study, where we found increased absolute QSM susceptibility values in Parkinson’s disease in the frontal and posterior parietal cortices.^31^

**Table 2:** Cortical regions with significant differences in QSM signal between patients with Parkinson’s disease and controls

ROI number	ROI name	HC, ppm	PD, ppm	*P*-value	*Q*-value
18	Fusiform face complex	9.44 × 10^−4^	3.02 × 10^−3^	0.025^*^	0.366
27	Precuneus visual area	−4.41 × 10^−3^	−1.43 × 10^−3^	0.016^*^	0.290
34	Area dorsal 23a+b	−5.90 × 10^−3^	−1.27 × 10^−3^	8.76 × 10^−4^^***^	0.079
37	Area 5 mm ventral	2.14 × 10^−3^	4.42 × 10^−3^	0.042^*^	0.447
38	Area 23c	−5.25 × 10^−3^	−3.01 × 10^−3^	0.028^*^	0.366
57	Area posterior 24 prime	−1.12 × 10^−2^	−8.39 × 10^−3^	0.014^*^	0.290
59	Anterior 24 prime	−9.01 × 10^−3^	−6.37 × 10^−3^	0.037^*^	0.443
73	Area 8 C	2.79 × 10^−3^	4.46 × 10^−3^	3.81 × 10^−3**^	0.229
83	Area posterior 9-46v	−2.01 × 10^−3^	−9.25 × 10^−4^	0.041^*^	0.447
85	Area anterior 9-46v	−6.73 × 10^−4^	6.27 × 10^−4^	9.71 × 10^−3**^	0.265
86	Area 9-46d	−3.48 × 10^−4^	7.59 × 10^−4^	0.028^*^	0.366
91	Area 11I	−4.68 × 10^−4^	1.21 × 10^−3^	7.63 × 10^−3**^	0.265
112	Anterior agranular insula complex	−6.48 × 10^−3^	−2.32 × 10^−3^	3.80 × 10^−5***^	6.85 × 10^−3**^
130	Area STSv posterior	−3.40 × 10^−3^	−1.23 × 10^−3^	0.015^*^	0.290
153	Ventromedial visual area 1	2.76 × 10^−3^	1.00 × 10^−4^	9.58 × 10^−3**^	0.265
167	Area posterior insular 1	−1.13 × 10^−2^	−8.34 × 10^−3^	0.018^*^	0.298
176	Area STSv anterior	5.67 × 10^−4^	2.83 × 10^−3^	0.010^*^	0.265

Region of interest numbers and names are given according to the Glasser atlas denomination. Age adjusted mean signed QSM values are reported. HC = healthy controls; PD = Parkinson’s disease; ROI = region of interest. *Q* represents the FDR-adjusted *P*. ***(*P* or *Q*) < 0.001; **(*P* or *Q*) < 0.01; *(*P* or *Q*) < 0.05; ns = not significant.

### Relating cortical brain iron in Parkinson’s disease to variation in gene expression patterns

We used PLS regression to identify the pattern of gene expression that correlated with the anatomical distribution of brain iron content, as measured using QSM. The PLS2 explained the most (20%) variance in the QSM score (*P* < 0.01) and the PLS2 gene expression weights showed a strong positive correlation with the QSM score (*r* = 0.44, *P* = 4.46 × 10^−^^10^, Fig. 3), meaning that genes that were positively weighted on PLS2 were also more highly expressed in cortical brain regions with higher magnetic susceptibilities. Similarly, genes that were negatively weighted on PLS2 showed relatively low expression in cortical brain regions with high QSM scores. The spatial profile of PLS2 weightings matched that of the QSM scores, particularly in the frontal, posterior cingulate and insular cortices. We therefore used PLS2 to rank and select significantly weighted genes, giving a set of 1622 significantly upweighted and 1068 significantly downweighted genes (*Q* < 0.05). We assessed these lists separately (Fig. 3). The complete set of PLS2 gene weights and associated statistics are provided in Supplementary Table 1.

**Figure 4:**
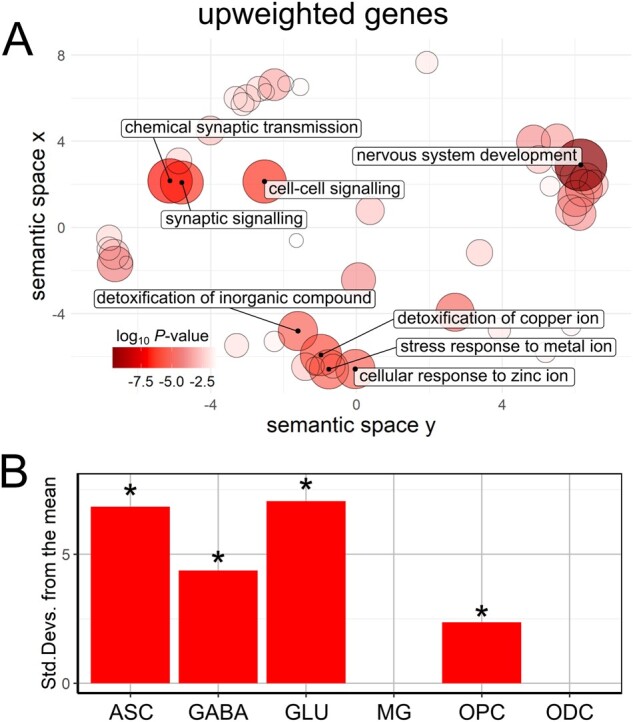
**Enrichment analyses for genes associated with cortical iron deposition in Parkinson’s disease.** (**A**) The gene ontological terms for biological processes that were significantly enriched in significantly upweighted genes defined by PLS2. The terms are plotted in semantic space with more similar terms clustered together. Non-redundant GO terms significant at g:SCS corrected *P* < 1 × 10^−5^ have been labelled in each case. Larger, darker circles indicate greater significance (see colour bar). (**B**) Expression-weighted cell-type enrichment analyses using the Allen Institute for Brain Science single-cell transcription dataset. Data are presented as standard deviations of the mean expression of upweighted target gene lists from the mean expression of the bootstrap replicates. Cell types in which the target gene lists are significantly enriched are marked with an asterisk (FDR corrected results). ASC = astrocytes; GABA = GABAergic neurons; GLU = glutamatergic neurons; MG = microglia; ODC = oligodendrocytes; OPC = oligodendrocyte precursor cells.

Using GO analyses, we found sets of biological processes associated with upweighted genes (Fig. 4A). Genes more highly expressed in regions with higher QSM values in Parkinson’s disease were enriched for GO terms relating to nervous system development, synaptic transmission and signalling and the detoxification of and stress response to metal ions (Table 3). We also found these genes to be enriched for the REACTOME pathways ‘Metallothioneins bind metals’ and ‘Response to metal ions’. In contrast, we did not find any GO biological processes to be enriched in the downweighted gene set. Full tables of the GO terms associated with up- and downweighted genes are provided in Supplementary Tables 2 and 3.

**Table 3:** Most significantly enriched GO biological processes in the upweighted gene list

Term ID	Term name	Term size	*P*-value
GO:0007399	Nervous system development	2483	1.53 × 10^−10^
GO:0099537	Trans-synaptic signalling	702	1.25 × 10^−7^
GO:0007268	Chemical synaptic transmission	694	1.32 × 10^−7^
GO:0098916	Anterograde trans-synaptic signalling	694	1.32 × 10^−7^
GO:0007267	Cell-cell signalling	1806	3.70 × 10^−7^
GO:0099536	Synaptic signalling	724	4.04 × 10^−7^
GO:0099177	Regulation of trans-synaptic signalling	421	1.06 × 10^−6^
GO:0010273	Detoxification of copper ion	14	1.57 × 10^−6^
GO:1990169	Stress response to copper ion	14	1.57 × 10^−6^
GO:0000902	Cell morphogenesis	1058	2.26 × 10^−6^
GO:0050804	Modulation of chemical synaptic transmission	420	2.60 × 10^−6^
GO:0097501	Stress response to metal ion	16	6.40 × 10^−6^
GO:0061687	Detoxification of inorganic compound	16	6.40 × 10^−6^
GO:0071294	Cellular response to zinc ion	23	6.73 × 10^−6^
GO:1902930	Regulation of alcohol biosynthetic process	83	1.38 × 10^−5^
GO:0022008	Neurogenesis	1710	2.57 × 10^−5^
GO:0048666	Neuron development	1167	2.90 × 10^−5^
GO:0071280	Cellular response to copper ion	27	4.78 × 10^−5^
GO:0031175	Neuron projection development	1030	5.28 × 10^−5^
GO:0006882	Cellular zinc ion homeostasis	37	7.43 × 10^−5^

The GO term ID is a unique gene ontology identifier; the term name gives a brief description; and the term size is the number of unique genes associated with a given term. The reported *P*-values are corrected for multiple comparisons using the g:SCS algorithm in g:Profiler.

Our additional control analyses confirmed that these findings were specific to the QSM data, rather than spuriously arising from gene-gene co-expression. Specifically, the control analysis using a spatial spin of the cortical data revealed upweighted genes that were not enriched for any GO biological processes, and downweighted genes enriched for biological processes relating to immune cell proliferation and viral transcription. See Supplementary Tables 4 and 5 for the full list of the GO terms associated with the up- and downweighted genes from the control spatial-spin null analyses. The second control analysis was performed using a random-null model and generated an upweighted gene list that was not significantly enriched for any GO biological processes and a downweighted list that was enriched only for the term ‘response to lipopolysaccharide’. These control analyses provide additional support that the gene lists arising from our experimental analysis relating spatial variation in QSM with gene expression are specific to this relationship and have not arisen spuriously due to gene-gene co-expression or spatial autocorrelation.

### Cell types linked with variation in brain cortical iron content

We assessed whether the most significantly upweighted and downweighted genes associated with brain iron, as measured using QSM, were more strongly expressed in specific cell types. Cell-type expression was defined using the human-derived single-nucleus data from the Allen Institute for Brain Science^58^ and increased expression assessed using expression-weighted cell-type enrichment analysis.^57^ We found that the top 20% of upweighted genes (324 genes) were significantly enriched in astrocytes, glutamatergic and GABAergic neurons and oligodendrocyte precursor cells, implying a relatively higher proportion of these cell types in healthy brains in regions with a higher iron contents in Parkinson’s disease (Fig. 4B). We found that the top 20% of downweighted genes (214 genes) showed significantly greater expression in GABAergic and glutamatergic neurons, implying a higher proportion of these cells in healthy brains in regions with lower iron contents in Parkinson’s disease. Adjusting the expression-weighted cell-type enrichment analysis threshold to include the top 10, 30, 40 or 50% of upweighted or downweighted genes did not alter the cell transcription profile seen in either case, nor did defining cell-type expression using a different human-derived single-cell nucleus dataset^59^ (Supplementary Fig. 1).

### Association with Parkinson’s disease post-mortem-derived gene expression data

We found that genes upregulated in Parkinson’s disease cortex relative to controls, as well as genes upregulated in cortex in Parkinson’s disease dementia relative to control subjects,^60^ were more negatively weighted than expected by chance in our analysis (*P* = 0.0002, *P* < 0.0001, respectively). Similarly, genes identified as being upregulated in cortex amongst individuals with dementia with Lewy bodies relative to controls^61^ were significantly more downweighted than expected by chance in our analysis (*P* < 0.0001), whereas genes found to be downregulated were significantly more upweighted (*P* < 0.0001). Additionally, we found that downregulated genes identified in Parkinson’s disease substantia nigra^61^ were more positively weighted than was due to chance in our analysis (*P* < 0.0001). Full results from the above weighting analysis can be found in Supplementary Table 8. Together, these results suggest that the gene expression profiles we identified based on susceptibility to cortical iron deposition and using baseline gene expression (as determined by the Allen Atlas dataset) are more perturbed in Parkinson’s disease than expected by chance, and this finding is replicable across independent sample cohorts and experimental approaches. Furthermore, these findings hold at later disease stages linked with Parkinson’s disease dementia and dementia with Lewy bodies.

## Discussion

We demonstrated that the regional increases in magnetic susceptibility that are most likely due to brain iron accumulation are associated with the expression of genes involved in metal detoxification and synaptic function. Regional differences in the probable proportion of astrocytes, glutamatergic and GABAergic neurons and oligodendrocyte precursor cells were also present.

Increased QSM susceptibility values were found in prefrontal and posterior parietal cortices, consistent with our previous findings,^31^ and were also present in insular and cingulate cortices. The use of signed rather than absolute QSM values allowed us to separate the effects of paramagnetic from diamagnetic substances, allowing for a more detailed analysis of the gene expression profiles. The cortical pattern of increased QSM values implicates similar cortical regions to those where Lewy pathology predominates in advanced Parkinson’s disease, namely the insular, cingulate and prefrontal cortices.^67^ High levels of intracellular iron promote α-synuclein fibril aggregation^23^^,^^68^ and produce free radical species causing cellular damage.^16–19^ We believe therefore that high QSM susceptibility in these regions is likely to reflect early tissue changes leading to neurodegeneration in Parkinson’s disease.

To connect the regional pattern of iron accumulation to underlying biological vulnerabilities, we used PLS regression to identify the pattern of gene expression that best mapped to the spatial distribution of the quantitative susceptibility mapping signal. Those genes with higher expression in regions with more brain iron deposition in Parkinson’s disease as measured by QSM (upweighted on PLS2) were significantly enriched for GO processes relating to heavy metal detoxification and synaptic signalling. The specific link between brain iron and cellular vulnerability in Parkinson’s disease was recently shown using a clustered regularly interspaced short palindromic repeats (CRISPR) interference platform. Mutations in genes related to lysosomal pathways, which are highly implicated in Parkinson’s disease, strongly sensitized neurons to oxidative stress and ferroptosis.^22^

Our finding of gene expression related to the detoxification of other heavy metals is intriguing. This is likely to be explained by the fact that iron-copper homeostasis is controlled by proteins involved in both the metabolism of iron and copper.^69^^,^^70^ As well as iron, altered levels of copper occur in Parkinson’s disease. Reduced copper is seen alongside increased iron in the substantia nigra in Parkinson’s disease,^8^ whereas increased copper has been reported in the CSF.^71^ Free copper can catalyse reactions generating reactive oxygen species in a manner similar to iron^72^ and has similar potential to cause cell damage. However, some copper binding and transport proteins, notably metallothioneins, may have a neuroprotective role.^73^ The altered binding of copper by cuproproteins in Parkinson’s disease could contribute to the inability of cells to deal with an increased oxidative load,^74^ which is in turn aggravated by a large pool of labile iron.^75^ Copper has a high binding affinity with α-synuclein and is more potent than iron in aggregating α-synuclein,^76^ as well as being implicated in the formation of amyloid-β plaques and neurofibrillary tangles.^77^ When bound to α-synuclein it also has the capacity to reduce ferric to ferrous iron, facilitating further reactive oxygen species.^78^

Disturbances in synaptic function are known to play a role in vulnerability and progression in Parkinson’s disease. A number genes mutated in familial forms of Parkinson’s disease are involved in synaptic function and autophagy,^79^ and genes implicated in sporadic Parkinson’s disease also relate to mechanisms of synaptic homeostasis.^80^ Aberrant synaptic autophagy and excess synaptic pruning may lead to downstream synaptic loss, which is expected to precede neurodegeneration.^80–82^

The distribution of gene expression could be explained partly by variation in the distribution of particular cell types, leading us to perform an expression-weighted cell-type enrichment analysis. We found that upweighted genes showed significantly greater expression in astrocytes and glutamatergic and GABAergic neurons and oligodendrocyte precursor cells. The finding of relative enrichment of astrocytes in regions with high levels of brain iron is intriguing, as astrocytes play an important role in brain iron uptake and metabolism^83^^,^^84^ and in distributing iron to other neuronal cells.^13^^,^^85^

Enrichment in glutamatergic neurons is also of note. Higher levels of brain iron have been reported to correlate with higher *N*-methyl-d-aspartate receptor overactivity^86^ and the increased activity of these receptors may also stimulate the release of iron from the lysosome.^87^ Glutamate-mediated excitotoxicity has been linked with neurodegeneration in Parkinson’s disease.^88^ Glutamate induces Parkin accumulation in mitochondria in a calcium and *N*-methyl-d-aspartate-receptor dependent manner,^89^ and α-synuclein may perturb intracellular calcium levels via α-amino-3-hydroxy-5-methyl-4-isoxazolepropionic acid (AMPA) receptors.^90^

We compared the pattern of cortical gene expression from our analyses against differentially expressed gene lists identified in a number of external post-mortem datasets in Parkinson’s disease, Parkinson’s disease dementia and dementia with Lewy bodies.^60–64^ We found that genes altered in these conditions were linked with the profile of healthy brain gene expression underlying higher magnetic susceptibility in Parkinson’s disease.^60^^,^^61^ This provides further evidence for processes involving brain iron in the selective vulnerabilities driving degeneration in Parkinson’s disease and Lewy body dementia.

Our findings demonstrate that regions showing increased iron deposition in Parkinson’s disease have a functional abundance of cells and proteins involved in the homeostasis and detoxification of metals in the healthy brain as well as in synaptic function. Any disturbance of such pathways, as has been reported in Parkinson’s disease and models of Parkinson’s disease, could potentially render these regions vulnerable to excess iron accumulation, aberrant iron processing and subsequent downstream oxidative stress, proteinopathy and cell death.

### Limitations

Our main analysis used gene expression data derived from donors unaffected by neurological or psychiatric disease. Spatial expression profiles are therefore based on unaffected individuals and conclusions only apply to this intrinsic variability, not to changes in gene expression that occur in Parkinson’s disease.

Because of the availability of data in the Allen Human Brain Atlas, we used only left hemisphere data, as data from the right hemisphere was only available from two brains.^32^^,^^48^ There is a lateralization effect for the distribution of QSM susceptibility, with higher magnetic susceptibilities in the right compared with the left hemisphere. Differences between hemispheres could be examined in future work when gene expression data are available for both hemispheres from a larger number of donors. When Parkinson’s disease gene expression data with widespread cortical coverage become available, it will be of interest to relate gene expression in Parkinson’s disease directly to brain iron levels. We were unable to validate our PLS analysis with any additional healthy gene expression databases as those currently available^91^^,^^92^ are derived from only a small number of cortical regions.

We used QSM to measure increased iron deposition in Parkinson’s disease and investigated the genetic makeup of implicated regions, as there is strong evidence implicating neurodegeneration as a process downstream of iron-induced oxidative stress.^9–13^ However, QSM is unable to capture all drivers of neurodegeneration, especially those that occur independently of iron accumulation or do not exhibit positive correlations with it. This may explain, for example, why we did not see any significant GO terms relating to autophagy or mitochondrial function in our analysis, despite reported associations between these two processes and Parkinson’s disease neurodegeneration.^93^^,^^94^ There may also be other drivers of QSM changes in magnetic susceptibility apart from iron. For example, it is possible that demyelination could lead to increases in cortical paramagnetic susceptibility,^95^ and diamagnetic metals such as calcium and magnesium have been reported at comparable levels to iron in several brain regions post-mortem.^96^

Iron deposits have been shown to co-localize with amyloid^97^ and tau pathology.^98^ How these contribute to one another and lead to neurodegeneration is not known. Future studies of this kind could incorporate contemporaneous data on brain amyloid and tau, e.g. using amyloid or tau PET, with QSM and transcriptomic data to shed light on the biological processes which may contribute to this.

Our cell-type analysis was performed using the Allen Institute for Brain Science single nucleus RNA-sequencing dataset, which is based on the profiling of cortical brain samples. Future analyses could specifically examine the role of cells in other brain regions, for example midbrain dopaminergic neurons.

Participants in our study were scanned whilst taking their usual dopaminergic medications. There is no evidence that l-DOPA causally affects brain tissue iron content or magnetic susceptibility, and l-DOPA does not alter cortical iron levels in a mouse model of Parkinson’s disease.^99^ In humans, magnetic susceptibility in subcortical regions of interest analyses do correlate with l-DOPA dose,^100^ but whether this relates specifically to the effects of l-DOPA or is a function of disease severity is not yet known. The effects of being ON versus OFF l-DOPA on cortical QSM magnetic susceptibility could specifically be examined in future work. Finally, our study was cross sectional. Longitudinal studies of brain iron accumulation in patients with Parkinson’s disease progressing to dementia will provide further insights into the temporal order of brain iron accumulation in specific brain regions and the associated biological processes.

## Conclusions

Regional increases in magnetic susceptibility in Parkinson’s disease, most likely due to brain iron accumulation in frontal, cingulate and insular cortices, are associated with a distinct gene expression profile. In these regions, we found higher intrinsic levels of gene expression relating to heavy metal detoxification and synaptic function as well as a probable relative abundance of astrocytes and glutamatergic neurons. These findings shed light on the processes driving neurodegeneration in Parkinson’s disease and the selective vulnerabilities of brain regions that are most affected, providing potential insights into future therapeutic targets to slow the progression of neurodegeneration in Parkinson’s disease.

## Funding

G.E.C.T. is supported by a PhD studentship from the Medical Research Council (MR/N013867/1). A.Z. is supported by an Alzheimer’s Research UK Clinical Research Fellowship (2018B-001). M.R. is supported is supported by an UK Medical Research Council Tenure-track Clinician Scientist Fellowship (MR/N008324/1). K.S. is supported by ERC Consolidator Grant DiSCo MRI SFN 770939. A.L.G.-M. is supported by Fundación Séneca (21230/PD/19). P.M. is supported by the National Institute for Health Research. R.S.W. is supported by a Wellcome Clinical Research Career Development Fellowship (205167/Z/16/Z). Recruitment to the study was also supported by Parkinson’s UK and the Cure Parkinson’s Trust. The study was further supported by UCLH Biomedical Research Centre Grant (BRC302/NS/RW/101410) and by grants from the National Institute for Health Research.

## Competing interests

R.S.W. has received personal fees from GE healthcare. J.A.-C. has equity and a full-time appointment at Tenoke Limited.

## Supplementary material

Supplementary material is available at *Brain* online.

## Supplementary Material

awab084_Supplementary_Data

## Abbreviations

QSM: quantitative susceptibility mapping

## References

[?>1] Hornykiewicz O. Dopamine miracle: From brain homogenate to dopamine replacement. Mov Disord. 2002;17:501-508. 12112197 10.1002/mds.10115

[?>2] Spillantini MG , Schmidt ML, Lee VMY, Trojanowski JQ, Jakes R, Goedert M. α-synuclein in Lewy bodies. Nature. 1997;388:839-840. 9278044 10.1038/42166

[?>3] Jellinger KA. A critical evaluation of current staging of α-synuclein pathology in Lewy body disorders. Biochim Biophys Acta Mol Basis Dis. 2009;1792:730-740. 10.1016/j.bbadis.2008.07.006 18718530

[?>4] Surmeier DJ , Obeso JA, Halliday GM. Selective neuronal vulnerability in Parkinson disease. Nat Rev Neurosci. 2017;18:101-113. 28104909 10.1038/nrn.2016.178 PMC5564322

[?>5] Johnson ME , Stecher B, Labrie V, Brundin L, Brundin P. Triggers, facilitators, and aggravators: Redefining Parkinson’s disease pathogenesis. Trends Neurosci. 2019;42:4-13. 30342839 10.1016/j.tins.2018.09.007 PMC6623978

[?>6] Dexter DT , Wells FR, Lees AJ, et al Increased nigral iron content and alterations in other metal ions occurring in brain in Parkinson’s disease. J Neurochem. 1989;52:1830-1836. 2723638 10.1111/j.1471-4159.1989.tb07264.x

[?>7] Sofic E , Riederer P, Heinsen H, et al Increased iron (III) and total iron content in post mortem substantia nigra of parkinsonian brain. J Neural Transm. 1988;74:199-205. 3210014 10.1007/BF01244786

[?>8] Dexter DT , Carayon A, Javoy-Agid F, et al Alterations in the levels of iron, ferritin and other trace metals in Parkinson’s disease and other neurodegenerative diseases affecting the basal ganglia. Brain. 1991;114:1953-1975. 1832073 10.1093/brain/114.4.1953

[?>9] Fahn S , Cohen G. The oxidant stress hypothesis in Parkinson’s disease: Evidence supporting it. Ann Neurol. 1992;32:804-812. 1471873 10.1002/ana.410320616

[10] Gerlach M , Ben-Shachar D, Riederer P, Youdim MBH. Altered brain metabolism of iron as a cause of neurodegenerative diseases? J Neurochem. 1994;63:793-807. 7519659 10.1046/j.1471-4159.1994.63030793.x

[11] Halliwell B. Reactive oxygen species and the central nervous system. J Neurochem. 1992;59:1609-1623. 1402908 10.1111/j.1471-4159.1992.tb10990.x

[12] Ndayisaba A , Kaindlstorfer C, Wenning GK. Iron in neurodegeneration - cause or consequence? Front Neurosci. 2019;13: 180. 30881284 10.3389/fnins.2019.00180 PMC6405645

[13] Ward RJ , Zucca FA, Duyn JH, Crichton RR, Zecca L. The role of iron in brain ageing and neurodegenerative disorders. Lancet Neurol. 2014;13:1045-1060. 25231526 10.1016/S1474-4422(14)70117-6 PMC5672917

[14] Betts MJ , Acosta-Cabronero J, Cardenas-Blanco A, Nestor PJ, Düzel E. High-resolution characterisation of the aging brain using simultaneous quantitative susceptibility mapping (QSM) and R2 measurements at 7 T. Neuroimage. 2016;138:43-63. 27181761 10.1016/j.neuroimage.2016.05.024

[15] Farrall AJ , Wardlaw JM. Blood-brain barrier: Ageing and microvascular disease - systematic review and meta-analysis. Neurobiol Aging. 2009;30:337-352. 17869382 10.1016/j.neurobiolaging.2007.07.015

[16] Horowitz MP , Greenamyre JT. Mitochondrial iron metabolism and its role in neurodegeneration. J Alzheimer’s Dis. 2010;20(Suppl 2):S551–S568. 10.3233/JAD-2010-100354 PMC3085540 20463401

[17] Melis JPM , Van Steeg H, Luijten M. Oxidative DNA damage and nucleotide excision repair. Antioxidants Redox Signal. 2013;18:2409-2419. 10.1089/ars.2012.5036 PMC3671630 23216312

[18] Hare DJ , Double KL. Iron and dopamine: A toxic couple. Brain. 2016;139:1026-1035. 26962053 10.1093/brain/aww022

[19] Dalle-Donne I , Giustarini D, Colombo R, Rossi R, Milzani A. Protein carbonylation in human diseases. Trends Mol Med. 2003;9:169-176. 12727143 10.1016/s1471-4914(03)00031-5

[20] Cozzi A , Orellana DI, Santambrogio P, et al Stem cell modeling of neuroferritinopathy reveals iron as a determinant of senescence and ferroptosis during neuronal aging. Stem Cell Reports. 2019;13:832-815. 31587993 10.1016/j.stemcr.2019.09.002 PMC6893074

[21] Braak E , Sandmann-Keil D, Rüb U, et al αSynuclein immunopositive Parkinson’s disease-related inclusion bodies in lower brain stem nuclei. Acta Neuropathol. 2001;101:195-201. 11307617 10.1007/s004010000247

[22] Tian R , Abarientos A, Hong J et al Genome-wide CRISPRi/a screens in human neurons link lysosomal failure to ferroptosis. *bioRxiv*. [Preprint] doi:10.1101/2020.06.27.175679

[23] Ostrerova-Golts N , Petrucelli L, Hardy J, Lee JM, Farer M, Wolozin B. The A53T alpha-synuclein mutation increases iron-dependent aggregation and toxicity. J Neurosci. 2000;20:6048-6054. 10934254 10.1523/JNEUROSCI.20-16-06048.2000 PMC6772599

[24] Silvestri L , Camaschella C. A potential pathogenetic role of iron in Alzheimer’s disease. J Cell Mol Med. 2008;12:1548-1550. 18466351 10.1111/j.1582-4934.2008.00356.x PMC3918070

[25] Huang X , Atwood CS, Hartshorn MA, et al The Aβ peptide of Alzheimer’s disease directly produces hydrogen peroxide through metal ion reduction. Biochemistry. 1999;38:7609-7616. 10386999 10.1021/bi990438f

[26] Lovell MA , Xiong S, Xie C, Davies P, Markesbery WR. Induction of hyperphosphorylated tau in primary rat cortical neuron cultures mediated by oxidative stress and glycogen synthase kinase-3. J Alzheimer’s Dis. 2004;6:659-671. 15665406 10.3233/jad-2004-6610

[27] Lanskey JH , McColgan P, Schrag AE, et al Can neuroimaging predict dementia in Parkinson’s disease? Brain. 2018;141:2545-2560. 30137209 10.1093/brain/awy211 PMC6113860

[28] Shmueli K , De Zwart JA, Van Gelderen P, Li TQ, Dodd SJ, Duyn JH. Magnetic susceptibility mapping of brain tissue in vivo using MRI phase data. Magn Reson Med. 2009;62:1510-1522. 19859937 10.1002/mrm.22135 PMC4275127

[29] Langkammer C , Schweser F, Krebs N, et al Quantitative susceptibility mapping (QSM) as a means to measure brain iron? A post mortem validation study. Neuroimage. 2012;62:1593-1599. 22634862 10.1016/j.neuroimage.2012.05.049 PMC3413885

[30] Sun H , Walsh AJ, Lebel RM, et al Validation of quantitative susceptibility mapping with Perls’ iron staining for subcortical gray matter. Neuroimage. 2015;105:486-492. 25462797 10.1016/j.neuroimage.2014.11.010

[31] Thomas GEC , Leyland LA, Schrag AE, Lees AJ, Acosta-Cabronero J, Weil RS. Brain iron deposition is linked with cognitive severity in Parkinson’s disease. J Neurol Neurosurg Psychiatry. 2020;91:418-425. 32079673 10.1136/jnnp-2019-322042 PMC7147185

[32] Hawrylycz MJ , Lein ES, Guillozet-Bongaarts AL, et al An anatomically comprehensive atlas of the adult human brain transcriptome. Nature. 2012;489:391-399. 22996553 10.1038/nature11405 PMC4243026

[33] Romero-Garcia R , Warrier V, Bullmore ET, Baron-Cohen S, Bethlehem RAI. Synaptic and transcriptionally downregulated genes are associated with cortical thickness differences in autism. Mol Psychiatry. 2019;24:1053-1064. 29483624 10.1038/s41380-018-0023-7 PMC6755982

[34] Morgan SE , Seidlitz J, Whitaker KJ, et al Cortical patterning of abnormal morphometric similarity in psychosis is associated with brain expression of schizophrenia-related genes. Proc Natl Acad Sci U S A. 2019;116:9604-9609. 31004051 10.1073/pnas.1820754116 PMC6511038

[35] McColgan P , Gregory S, Seunarine KK, et al; Track-On HD Investigators. Brain regions showing white matter loss in Huntington’s disease are enriched for synaptic and metabolic genes. Biol Psychiatry. 2018;83:456-465. 29174593 10.1016/j.biopsych.2017.10.019 PMC5803509

[36] Gibb WRG , Lees AJ. The relevance of the Lewy body to the pathogenesis of idiopathic Parkinson’s disease. J Neurol Neurosurg Psychiatry. 1988;51:745-752. 2841426 10.1136/jnnp.51.6.745 PMC1033142

[37] Goetz CG , Tilley BC, Shaftman SR, Stebbins GT, et al; Movement Disorder Society UPDRS Revision Task Force. Movement disorder society-sponsored revision of the unified parkinson’s disease rating scale (MDS-UPDRS): Scale presentation and clinimetric testing results. Mov Disord. 2008;23:2129-2170. 19025984 10.1002/mds.22340

[38] Folstein MF , Folstein SE, McHugh PR. ‘ Mini-mental state’. A practical method for grading the cognitive state of patients for the clinician. J Psychiatr Res. 1975;12:189-198. 1202204 10.1016/0022-3956(75)90026-6

[39] Nasreddine ZS , Phillips NA, Bédirian V, et al The Montreal Cognitive Assessment, MoCA: A brief screening tool for mild cognitive impairment. J Am Geriatr Soc. 2005;53:695-699. 15817019 10.1111/j.1532-5415.2005.53221.x

[40] Abdul-Rahman HS , Gdeisat MA, Burton DR, Lalor MJ, Lilley F, Moore CJ. Fast and robust three-dimensional best path phase unwrapping algorithm. Appl Opt. 2007;46:6623-6635. 17846656 10.1364/ao.46.006623

[41] Smith SM. Fast robust automated brain extraction. Hum Brain Mapp. 2002;17:143-155. 12391568 10.1002/hbm.10062 PMC6871816

[42] Zhou D , Liu T, Spincemaille P, Wang Y. Background field removal by solving the Laplacian boundary value problem. NMR Biomed. 2014;27:312-319. 24395595 10.1002/nbm.3064

[43] Schweser F , Deistung A, Lehr BW, Reichenbach JR. Quantitative imaging of intrinsic magnetic tissue properties using MRI signal phase: An approach to in vivo brain iron metabolism? Neuroimage. 2011;54:2789-2807. 21040794 10.1016/j.neuroimage.2010.10.070

[44] Acosta-Cabronero J , Milovic C, Mattern H, Tejos C, Speck O, Callaghan MF. A robust multi-scale approach to quantitative susceptibility mapping. Neuroimage. 2018;183:7-24. 30075277 10.1016/j.neuroimage.2018.07.065 PMC6215336

[45] Avants BB , Tustison NJ, Song G, Cook PA, Klein A, Gee JC. A reproducible evaluation of ANTs similarity metric performance in brain image registration. Neuroimage. 2011;54:2033-2044. 20851191 10.1016/j.neuroimage.2010.09.025 PMC3065962

[46] Acosta-Cabronero J , Cardenas-Blanco A, Betts MJ, et al The whole-brain pattern of magnetic susceptibility perturbations in Parkinson’s disease. Brain. 2017;140:118-131. 27836833 10.1093/brain/aww278

[47] Glasser MF , Coalson TS, Robinson EC, et al A multi-modal parcellation of human cerebral cortex. Nature. 2016;536:171-178. 27437579 10.1038/nature18933 PMC4990127

[48] Arnatkevic˘iūtė A , Fulcher BD, Fornito A. A practical guide to linking brain-wide gene expression and neuroimaging data. Neuroimage. 2019;189:353-367. 30648605 10.1016/j.neuroimage.2019.01.011

[49] Yekutieli D , Benjamini Y. Resampling-based false discovery rate controlling multiple test procedures for correlated test statistics. J Stat Plan Inference. 1999;82:171-196.

[50] Romme IAC , de Reus MA, Ophoff RA, Kahn RS, van den Heuvel MP. Connectome disconnectivity and cortical gene expression in patients with schizophrenia. Biol Psychiatry. 2017;81:495-502. 27720199 10.1016/j.biopsych.2016.07.012

[51] Abdi H. Partial least squares regression and projection on latent structure regression (PLS Regression). Wiley Interdiscip Rev Comput Stat. 2010;2:97-106.

[52] Váša F , Seidlitz J, Romero-Garcia R, et al; NSPN consortium. Adolescent tuning of association cortex in human structural brain networks. Cereb Cortex. 2018;28:281-294. 29088339 10.1093/cercor/bhx249 PMC5903415

[53] Bigdeli TB , Lee D, Webb BT, et al A simple yet accurate correction for winner’s curse can predict signals discovered in much larger genome scans. Bioinformatics. 2016;32: 2598-2603. 27187203 10.1093/bioinformatics/btw303 PMC5013908

[54] Raudvere U , Kolberg L, Kuzmin I, et al g: Profiler: A web server for functional enrichment analysis and conversions of gene lists (2019 update). Nucleic Acids Res. 2019;47:191-198. 10.1093/nar/gkz369 PMC6602461 31066453

[55] Supek F , Bošnjak M, Škunca N, Šmuc T. Revigo summarizes and visualizes long lists of gene ontology terms. PLoS One. 2011;6:e21800. 21789182 10.1371/journal.pone.0021800 PMC3138752

[56] Fulcher BD , Arnatkevičiūtė A, Fornito A. Overcoming bias in gene-set enrichment analyses of brain-wide transcriptomic data. *bioRxiv*. [Preprint] doi:10.1101/2020.04.24.058958

[57] Skene NG , Grant SGN. Identification of vulnerable cell types in major brain disorders using single cell transcriptomes and expression weighted cell type enrichment. Front Neurosci. 2016;10:16. 26858593 10.3389/fnins.2016.00016 PMC4730103

[58] Hawrylycz M , Miller JA, Menon V, et al Canonical genetic signatures of the adult human brain. Nat Neurosci. 2015;18:1832-1844. 26571460 10.1038/nn.4171 PMC4700510

[59] Habib N , Avraham-Davidi I, Basu A, et al Massively parallel single-nucleus RNA-seq with DroNc-seq. Nat Methods. 2017;14:955-958. 28846088 10.1038/nmeth.4407 PMC5623139

[60] Dumitriu A , Golji J, Labadorf AT, Gao B, et al Integrative analyses of proteomics and RNA transcriptomics implicate mitochondrial processes, protein folding pathways and GWAS loci in Parkinson disease. BMC Med Genomics. 2016;9:5. 26793951 10.1186/s12920-016-0164-y PMC4722694

[61] Riley BE , Gardai SJ, Emig-Agius D, et al Systems-based analyses of brain regions functionally impacted in Parkinson’s disease reveals underlying causal mechanisms. PLoS One. 2014;9:e102909. 25170892 10.1371/journal.pone.0102909 PMC4149353

[62] Stamper C , Siegel A, Liang WS, et al Neuronal gene expression correlates of Parkinson’s disease with dementia. Mov Disord. 2008;23:1588-1595. 18649390 10.1002/mds.22184 PMC2666445

[63] Bossers K , Meerhoff G, Balesar R, et al Analysis of gene expression in Parkinson’s disease: Possible involvement of neurotrophic support and axon guidance in dopaminergic cell death. Brain Pathol. 2009;19:91-107. 18462474 10.1111/j.1750-3639.2008.00171.x PMC8094761

[64] Dijkstra AA , Ingrassia A, De Menezes RX, et al Evidence for immune response, axonal dysfunction and reduced endocytosis in the substantia nigra in early stage Parkinson’s disease. PLoS One. 2015;10:e0128651-21. 26087293 10.1371/journal.pone.0128651 PMC4472235

[65] Whitaker KJ , Vértes PE, Romero-Garciaa R, et al; NSPN Consortium. Adolescence is associated with genomically patterned consolidation of the hubs of the human brain connectome. Proc Natl Acad Sci U S A. 2016;113:9105-9110. 27457931 10.1073/pnas.1601745113 PMC4987797

[66] Xia M , Wang J, He Y. BrainNet viewer: A network visualization tool for human brain connectomics. PLoS One. 2013;8:e68910. 23861951 10.1371/journal.pone.0068910 PMC3701683

[67] Braak H , Del Tredici K, Rüb U, De Vos RAI, Jansen Steur ENH, Braak E. Staging of brain pathology related to sporadic Parkinson’s disease. Neurobiol Aging. 2003;24:197-211. 12498954 10.1016/s0197-4580(02)00065-9

[68] Li W , Jiang H, Song N, Xie J. Oxidative stress partially contributes to iron-induced alpha-synuclein aggregation in SK-N-SH cells. Neurotox Res. 2011;19:435-442. 20383623 10.1007/s12640-010-9187-x

[69] Kozlowski H , Luczkowski M, Remelli M, Valensin D. Copper, zinc and iron in neurodegenerative diseases (Alzheimer’s, Parkinson’s and prion diseases). Coord Chem Rev. 2012;256:2129-2141.

[70] Zheng W , Monnot AD. Regulation of brain iron and copper homeostasis by brain barrier systems: Implication in neurodegenerative diseases. Pharmacol Ther. 2012;133:177-188. 22115751 10.1016/j.pharmthera.2011.10.006 PMC3268876

[71] Boll MC , Sotelo J, Otero E, Alcaraz-Zubeldia M, Rios C. Reduced ferroxidase activity in the cerebrospinal fluid from patients with Parkinson’s disease. Neurosci Lett. 1999;265:155-158. 10327154 10.1016/s0304-3940(99)00221-9

[72] Wang H , Wang M, Wang B, et al The distribution profile and oxidation states of biometals in APP transgenic mouse brain: Dyshomeostasis with age and as a function of the development of Alzheimer’s disease. Metallomics. 2012;4:289-296. 22301945 10.1039/c2mt00104g

[73] Michael GJ , Esmailzadeh S, Moran LB, Christian L, Pearce RKB, Graeber MB. Up-regulation of metallothionein gene expression in Parkinsonian astrocytes. Neurogenetics. 2011;12:295-305. 21800131 10.1007/s10048-011-0294-5

[74] Montes S , Rivera-Mancia S, Diaz-Ruiz A, Tristan-Lopez L, Rios C. Copper and copper proteins in Parkinson’s disease. Oxid Med Cell Longev. 2014;2014:147251. 24672633 10.1155/2014/147251 PMC3941957

[75] Genoud S , Roberts BR, Gunn AP, et al Subcellular compartmentalisation of copper, iron, manganese, and zinc in the Parkinson’s disease brain. Metallomics. 2017;9:1447-1455. 28944802 10.1039/c7mt00244k PMC5647261

[76] Binolfi A , Quintanar L, Bertoncini CW, Griesinger C, Fernández CO. Bioinorganic chemistry of copper coordination to alpha-synuclein: Relevance to Parkinson’s disease. Coord Chem Rev. 2012;256:2188-2201.

[77] Sayre LM , Perry G, Harris PLR, Liu Y, Schubert KA, Smith MA. In situ oxidative catalysis by neurofibrillary tangles and senile plaques in Alzheimer’s disease: A central role for bound transition metals. J Neurochem. 2000;74(1):270-9. 10617129 10.1046/j.1471-4159.2000.0740270.x

[78] Davies P , Moualla D, Brown DR. Alpha-synuclein is a cellular ferrireductase. PLoS One. 2011;6(1):e15814. 10.1371/journal.pone.0015814 PMC3018422 21249223

[79] Kumaran R , Cookson MR. Pathways to Parkinsonism redux: Convergent pathobiological mechanisms in genetics of Parkinson’s disease. Hum Mol Genet. 2015;24:R32-R44. 26101198 10.1093/hmg/ddv236 PMC4571999

[80] Soukup S , Vanhauwaert R, Verstreken P. Parkinson’s disease: Convergence on synaptic homeostasis. EMBO J. 2018;37:1-16. 30065071 10.15252/embj.201898960 PMC6138432

[81] Day M , Wang Z, Ding J, et al Selective elimination of glutamatergic synapses on striatopallidal neurons in Parkinson disease models. Nat Neurosci. 2006;9:251-259. 16415865 10.1038/nn1632

[82] Plowey ED , Chu CT. Synaptic dysfunction in genetic models of Parkinson’s disease: A role for autophagy? Neurobiol Dis. 2011;43:60-67. 20969957 10.1016/j.nbd.2010.10.011 PMC3049988

[83] Abbott NJ , Rönnbäck L, Hansson E. Astrocyte-endothelial interactions at the blood-brain barrier. Nat Rev Neurosci. 2006;7:41-53. 16371949 10.1038/nrn1824

[84] Dringen R , Bishop GM, Koeppe M, Dang TN, Robinson SR. The pivotal role of astrocytes in the metabolism of iron in the brain. Neurochem Res. 2007;32:1884-1890. 17551833 10.1007/s11064-007-9375-0

[85] Jeong SY , David S. Glycosylphosphatidylinositol-anchored ceruloplasmin is required for iron efflux from cells in the central nervous system. J Biol Chem. 2003;278:27144-27148. 12743117 10.1074/jbc.M301988200

[86] Xu H , Jiang H, Xie J. New insights into the crosstalk between NMDARs and iron: Implications for understanding pathology of neurological diseases. Front Mol Neurosci. 2017;10:1-10. 28360837 10.3389/fnmol.2017.00071 PMC5352910

[87] White RS , Bhattacharya AK, Chen Y, et al Lysosomal iron modulates NMDA receptor-mediated excitation via small GTPase, Dexras1. Mol Brain. 2016;9: 38. 27080392 10.1186/s13041-016-0220-8 PMC4832449

[88] Ambrosi G , Cerri S, Blandini F. A further update on the role of excitotoxicity in the pathogenesis of Parkinson’s disease. J Neural Transm. 2014;121:849-859. 24380931 10.1007/s00702-013-1149-z

[89] Van Laar VS , Roy N, Liu A, et al Glutamate excitotoxicity in neurons triggers mitochondrial and endoplasmic reticulum accumulation of Parkin, and, in the presence of N-acetyl cysteine, mitophagy. Neurobiol Dis. 2015;74:180-193. 25478815 10.1016/j.nbd.2014.11.015 PMC4322770

[90] Hüls S , Högen T, Vassallo N, et al AMPA-receptor-mediated excitatory synaptic transmission is enhanced by iron-induced α-synuclein oligomers. J Neurochem. 2011;117:868-878. 21426349 10.1111/j.1471-4159.2011.07254.x

[91] Kang HJ , Kawasawa YI, Cheng F, et al Spatio-temporal transcriptome of the human brain. Nature. 2011;478:483-489. 22031440 10.1038/nature10523 PMC3566780

[?>92] Ramasamy A , Trabzuni D, Guelfi S, et al; North American Brain Expression Consortium. Genetic variability in the regulation of gene expression in ten regions of the human brain. Nat Neurosci. 2014;17:1418-1428. 25174004 10.1038/nn.3801 PMC4208299

[?>93] Menzies FM , Fleming A, Caricasole A, et al Autophagy and neurodegeneration: Pathogenic mechanisms and therapeutic opportunities. Neuron. 2017;93:1015-1034. 28279350 10.1016/j.neuron.2017.01.022

[?>94] Tan EK , Chao YX, West A, Chan LL, Poewe W, Jankovic J. Parkinson disease and the immune system—associations, mechanisms and therapeutics. Nat Rev Neurol. 2020;16:303-318. 32332985 10.1038/s41582-020-0344-4

[?>95] Stüber C , Morawski M, Schäfer A, et al Myelin and iron concentration in the human brain: A quantitative study of MRI contrast. Neuroimage. 2014;93:95-106. 24607447 10.1016/j.neuroimage.2014.02.026

[?>96] Krebs N , Langkammer C, Goessler W, et al Assessment of trace elements in human brain using inductively coupled plasma mass spectrometry. J Trace Elem Med Biol. 2014;28:1-7. 24188895 10.1016/j.jtemb.2013.09.006

[?>97] van Bergen JMG , Li X, Hua J, et al Colocalization of cerebral iron with amyloid beta in mild cognitive impairment. Sci Rep. 2016;6:1-9. 27748454 10.1038/srep35514 PMC5066274

[?>98] Spotorno N , Acosta-Cabronero J, Stomrud E, et al Relationship between cortical iron and tau aggregation in Alzheimer’s disease. Brain. 2020;143:1341-1349. 32330946 10.1093/brain/awaa089 PMC7241946

[?>99] Billings JL , Gordon SL, Rawling T, et al l-3,4-dihydroxyphenylalanine (l-DOPA) modulates brain iron, dopaminergic neurodegeneration and motor dysfunction in iron overload and mutant alpha-synuclein mouse models of Parkinson’s disease. J Neurochem. 2019;150:88-106. 30716176 10.1111/jnc.14676

[100] Langkammer C , Pirpamer L, Seiler S, et al Quantitative susceptibility mapping in Parkinson’s disease. PLoS One. 2016;11:e0162460. 27598250 10.1371/journal.pone.0162460 PMC5012676

