# Supplementary material for: Regional brain iron and gene expression provide insights into neurodegeneration in Parkinson’s disease
Source: Brain. 2021 Mar 11;144(6):1787–98. doi: 10.1093/brain/awab084 (PMC8320305; doi:10.1093/brain/awab084)
Supplement: awab084_Supplementary_Data [file awab084_supplementary_data.zip › awab084-suppl_data/brain-2020-01802-File015.docx]

| **Study** | **N subjects** | **Sample region** | **Expression** | **Matched genes**  **(all genes)** | **Mean weighting difference** | **P value** |
| --- | --- | --- | --- | --- | --- | --- |
|  |  |  |  |  |  |  |
| Stamper *et al*. 2008 | 15 PD, 14 PDD, 14 HC | PCC | PD > HC | 10 (24) | **-1.619** | **0.0033** |
|  |  |  | PD < HC | 35 (76) | -0.041 | 0.4599 |
|  |  |  | PDD > PD + HC | 14 (35) | **-0.945** | **0.0294** |
|  |  |  | PDD < PD + HC | 32 (65) | -0.225 | 0.2520 |
|  |  |  |  |  |  |  |
| Bossers *et al.* 2009 | 4 PD, 4 HC | SN | PD > HC | 29 (47) | +0.360 | 0.1516 |
|  |  |  | PD < HC | 178 (240) | **+0.342** | **0.0079** |
|  |  |  |  |  |  |  |
| Riley *et al.* 2014 | 16 PD, 20 HC | Cortex | PD > HC | 48 (63) | **+0.719** | **0.0036** |
|  |  |  | PD < HC | 132 (193) | **-0.391** | **0.0079** |
|  | 17 PD, 17 HC | Putamen | PD > HC | 116 (131) | **+0.326** | **0.0305** |
|  |  |  | PD < HC | 36 (38) | -0.027 | 0.4620 |
|  | 16 PD, 14 HC | SN | PD > HC | 77 (88) | +0.309 | 0.0738 |
|  |  |  | PD < HC | 166 (186) | **+0.106** | **<0.0001** |
|  | 3 DLB, 3 HC | Cortex | DLB > HC | 179 (202) | **-0.541** | **<0.0001** |
|  |  |  | DLB < HC | 399 (477) | **+0.863** | **<0.0001** |
|  |  |  |  |  |  |  |
| Dijkstra *et al.* 2015 | 9 PD, 8 HC | SN | PD > HC | 359 (583) | -0.132 | 0.0866 |
|  |  |  | PD < HC | 428 (826) | +0.068 | 0.2115 |
|  |  |  |  |  |  |  |
| Dumitriu *et al.* 2016* | 18 PD, 11 PDD, 44 HC | BA6 | PD > HC | 103 (334) | **-0.770** | **<0.0001** |
|  |  |  | PD < HC | 193 (420) | +0.178 | 0.0957 |
|  |  |  | PDD > HC | 191 (783) | **-0.486** | **0.0002** |
|  |  |  | PDD < HC | 291 (718) | +0.107 | 0.1660 |
|  |  |  | PDD > PD | 88 (329) | **+0.509** | **0.0055** |
|  |  |  | PDD < PD | 100 (286) | +0.295 | 0.0549 |
|  |  |  |  |  |  |  |
| **Supplementary Table 6 – Weighting of differentially expressed genes from external datasets in PLS2.** BA6 = Brodmann area 6; DLB = dementia with Lewy bodies; HC = healthy controls; PCC = posterior cingulate cortex; PD = Parkinson’s disease; PDD = Parkinson’s disease dementia; SN = substantia nigra. All differentially expressed genes included here have an absolute fold change of 1.5 or greater. P-values in bold are significant at p<0.05, P-values in bold underlined are significant after Bonferroni correction for multiple comparisons.  * This dataset was re-analysed using participant cognitive data to differentiate between likely PD and PDD subgroups. | | | | | | |
