# Supplementary figures and images for: Regional brain iron and gene expression provide insights into neurodegeneration in Parkinson’s disease
Source: Brain. 2021 Mar 11;144(6):1787–98. doi: 10.1093/brain/awab084 (PMC8320305; doi:10.1093/brain/awab084)

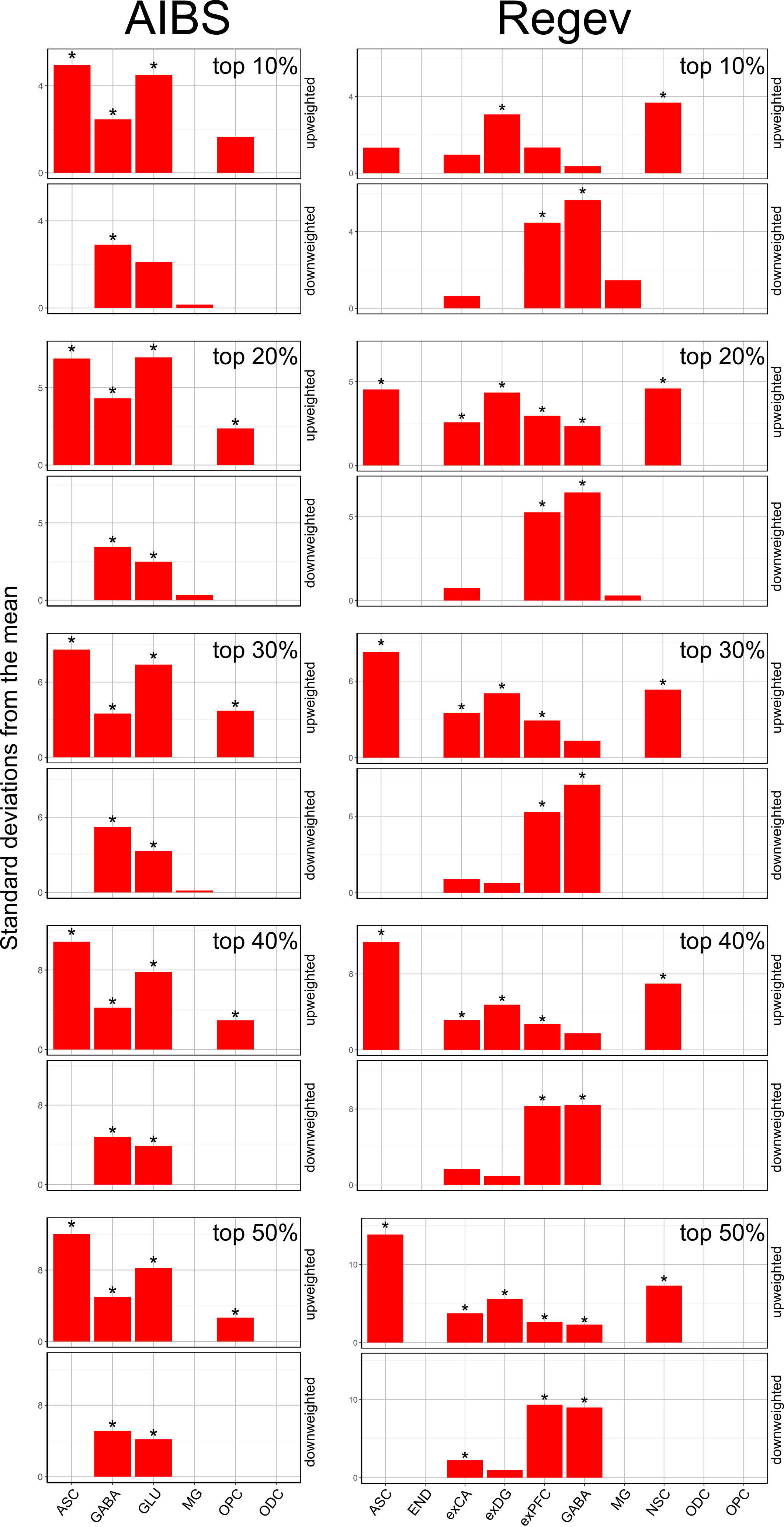

Supplement: awab084_Supplementary_Data [file awab084_supplementary_data.zip › awab084-suppl_data/brain-2020-01802-File009.png]
